# Mating competitiveness of sterile genetic sexing strain males (GAMA) under laboratory and semi-field conditions: Steps towards the use of the Sterile Insect Technique to control the major malaria vector *Anopheles arabiensis* in South Africa

**DOI:** 10.1186/s13071-016-1385-9

**Published:** 2016-03-02

**Authors:** Givemore Munhenga, Basil D. Brooke, Jeremie R. L. Gilles, Kobus Slabbert, Alan Kemp, Leonard C. Dandalo, Oliver R. Wood, Leanne N. Lobb, Danny Govender, Marius Renke, Lizette L. Koekemoer

**Affiliations:** Centre for Opportunistic, Tropical and Hospital Infections, National Institute for Communicable Diseases, Private Bag X4, Sandringham, Johannesburg, South Africa; Wits Research Institute for Malaria, School of Pathology, Faculty of Health Sciences, University of the Witwatersrand, Johannesburg, South Africa; Insect Pest Control Laboratory, Joint FAO/IAEA Division of Nuclear Techniques in Food and Agriculture, International Atomic Energy Agency, Vienna, Austria; iThemba LABS (Laboratory for Accelerator Based Sciences), Somerset West, South Africa; Special Pathogens Unit, Center for Opportunistic, Tropical and Hospital Infections, National Institute for Communicable Diseases, Private Bag X4, Sandringham, Johannesburg South Africa; Scientific Services, South African National Parks, Private Bag X402, Skukuza, South Africa; Department of Paraclinical Sciences, Faculty of Veterinary Science, University of Pretoria, Private Bag X04, Onderstepoort, South Africa; Conservation Management, Kruger National Park, Private Bag X402, Skukuza, South Africa

**Keywords:** Malaria, Vector control, Sterile insect technique, Mating competitiveness, *Anopheles arabiensis*, South Africa

## Abstract

**Background:**

*Anopheles arabiensis* Patton is primarily responsible for malaria transmission in South Africa after successful suppression of other major vector species using indoor spraying of residual insecticides. Control of *An. arabiensis* using current insecticide based approaches is proving difficult owing to the development of insecticide resistance, and variable feeding and resting behaviours. The use of the sterile insect technique as an area-wide integrated pest management system to supplement the control of *An. arabiensis* was proposed for South Africa and is currently under investigation. The success of this technique is dependent on the ability of laboratory-reared sterile males to compete with wild males for mates. As part of the research and development of the SIT technique for use against *An. arabiensis* in South Africa, radio-sensitivity and mating competitiveness of a local *An. arabiensis* sexing strain were assessed.

**Methods:**

The optimal irradiation dose inducing male sterility without compromising mating vigour was tested using Cobalt 60 irradiation doses ranging from 70-100 Gy. Relative mating competitiveness of sterile laboratory-reared males (GAMA strain) compared to fertile wild-type males (AMAL strain) for virgin wild-type females (AMAL) was investigated under laboratory and semi-field conditions using large outdoor cages. Three different sterile male to fertile male to wild-type female ratios were evaluated [1:1:1, 5:1:1 and 10:1:1 (sterile males: fertile, wild-type males: fertile, wild-type females)].

**Results:**

Irradiation at the doses tested did not affect adult emergence but had a moderate effect on adult survivorship and mating vigour. A dose of 75 Gy was selected for the competitiveness assays. Mating competitiveness experiments showed that irradiated GAMA male mosquitoes are a third as competitive as their fertile AMAL counterparts under semi-field conditions. However, they were not as competitive under laboratory conditions. An inundative ratio of 10:1 induced the highest sterility in the representative wild-type population, with potential to effectively suppress reproduction.

**Conclusion:**

Laboratory-reared and sterilised GAMA male *An. arabiensis* at a release ratio of 3:1 (3 sterile males to 1 wild, fertile male) can successfully compete for insemination of wild-type females. These results will be used to inform subsequent small-scale pilot field releases in South Africa.

## Background

South Africa has made significant progress towards reducing its malaria burden. The disease is now limited to northern KwaZulu-Natal, eastern Mpumalanga and north-eastern Limpopo provinces [1]. In these areas, malaria is endemic and characterised by unstable seasonal transmission maintained by *Anopheles arabiensis* [1]. The success of malaria control and near elimination of the other major vector species in South Africa, namely *An. funestus*, can be attributed to sustained vector control efforts which have been in operation since the 1940’s [2]. These efforts depend on indoor residual spraying (IRS) of households with either DDT (in traditional mud-walled houses) or synthetic pyrethroids (in modern cement-brick houses) [3]. Although IRS has created malaria free zones in most parts of the country, its efficacy can be undermined by a variety of reasons one of which is the development of insecticide resistance in target vector populations [4–6]. IRS predominantly targets indoor biting and resting mosquitoes [7] and cannot control those vectors that prefer outdoor feeding and resting, such as *An. arabiensis*. This species has recently been implicated in residual (outdoor) transmission in northern KwaZulu-Natal (Dandalo et al., unpublished data). As South Africa moves towards malaria elimination, complementary vector control strategies that address the problem of insecticide resistance, are environmentally friendly and are geared toward targeting outdoor biting sectors of vector populations, need to be explored. The use of the Sterile Insect Technique (SIT) has been proposed for this purpose and is under investigation in South Africa [8, 9].

SIT is an area wide form of pest management which is based on the mass production and release of sterile males to mate with indigenous females [10]. Sustained releases of sterile males affect reproductive capacity of the wild females resulting in population suppression and in some instances eradication of the target population [11]. The most successful application of this technique was the elimination of the new-world screwworm fly, *Cochliomyia hominivorax*, from the Americas in the 1950’s [12]. Since then the SIT has been applied to the control of a variety of insect species [13–15]. Continued success of SIT in agricultural pest species control, coupled with the development of new technologies and improvements in mosquito rearing methods has seen renewed interest in the use of this technique for mosquito vector control [16–18].

A primary drawback of SIT as an insect control strategy is the challenge of developing a laboratory strain which is both reproductively compatible and competitive with the targeted population. The processes involved in the development of a strain for SIT range from colonisation, development of a gender separation (sexing) system, optimisation of a sterilisation/irradiation system and development of mass production systems. [19] These processes can alter the genotypes and corresponding phenotypes in an insect strain [20] which may lead to mating incompatibility with the targeted population and or reduced mating competitiveness under field settings. Colonisation tends to select for those individuals whose genomes are best suited to proliferation under laboratory conditions [21] and is usually accompanied by bottle-necking and founder effect which reduces genetic variation and consequently leading to altered mating characteristics that compromise mating under field conditions [22, 23]. In mosquitoes mating behaviours involve a complex interplay of various factors including light intensity, circadian rhythm and location of physical swarm markers [24, 25]. Due to confined spaces experienced under artificial insectary conditions during colonisation these factors cannot be sufficiently mimicked resulting in colonised mosquitoes losing their ability to mate under natural conditions. In addition to colonisation, development of a sexing system, regardless of the approach used involves complex physical and genetic manipulations that can affect fitness and mating competitiveness. For example, the physical stresses imposed during the mechanical sifting of Mediterranean fruit fly pupae caused reduced male quality [26]. In mosquitoes, the complex chromosomal translocations involved in the creation of a genetic sexing strain have been shown to have a deleterious effect on male mating competitiveness [27, 28]. Lastly, the induction of sterility using ionising radiation may also compromise fitness and mating competitiveness [29].

Within the framework of developing SIT as complementary malaria vector control tool in South Africa, a local *An. arabiensis* strain was colonised using the progeny of wild females collected from the Kruger National Park. This strain has been maintained for five years to date and has been periodically infused with wild-collected material. Recently, an *An. arabiensis* genetic sexing strain (GSS), ANO IPCL1 [30] has been introgressed into the local South African strain. The ANO IPCL1 strain is characterised by the Y-linked dieldrin resistance gene and this sex-linked dieldrin resistance mechanism was successfully transferred into the local GSS strain during the introgression process (Munhenga, unpublished data). Successful use of this strain for SIT releases will largely depend on the comparative mating performance of irradiated, colonised males. The aim of this study was therefore to evaluate the relative mating competitiveness of laboratory-reared and sterilised males drawn from the local genetic sexing strain in a local setting and context, as well as to determine the optimal irradiation dose inducing complete male sterility in the newly established strain.

## Methods

### Study site

Dose optimisation and laboratory competitive assays were carried out under standard insectary conditions in the Botha DeMeillon insectary, Vector Control Reference Laboratory (VCRL) of the National Institute for Communicable Diseases (NICD), Johannesburg, South Africa. The rearing conditions were 25 °C, 85 % relative humidity and a photo period of 12:12 hour light/darkness, with a 45-min dawn and dusk light regimen. Field mating competitiveness experiments were carried out in large (walk-in) field cages under natural environmental conditions at Louis Se Gat, Kruger National Park, South Africa, between February and May 2014, which corresponds to the end of rainy / summer season. Louis Se Gat (23°06’39.88”S, 31°27’24.90”E) is located in the northern Kruger National Park. The site is surrounded by trees including *Croton megalobotrys* (Large fever berry), *Acacia robusta*, *Acacia xanthoploea* (Fever tree), *Loncho carpus capassa* (Apple leaf), and *Combretum imberbe* (Lead wood) that shaded the field cages during the competitiveness assays.

### Mosquito strains

Two mosquito strains were used during this study. AMAL is a representative *An. arabiensis* wild-type strain originating from material collected at Malahlapanga in the Kruger National Park [9]. AMAL has been maintained in colony since 2010 with periodic revitalisations using field-collected material. Another strain, GAMA, is a genetic sexing strain (GSS) which was developed by introgressing AMAL females with GSS ANO IPCL1 males (provided by the FAO/IAEA Insect Pest Laboratory (IPCL), Seibersdorf, Austria) carrying dieldrin resistance on the Y-chromosome (***G****SS* X ***AMA****L*). The resultant offspring were backcrossed to AMAL females to produce a strain where males carry dieldrin resistance on the Y-chromosome in a genotypic background representing the South African population. Both strains are maintained in the Botha DeMeillon insectary, Vector Control Reference Laboratory (VCRL) of the National Institute for Communicable Diseases (NICD), Johannesburg, South Africa, under standard insectary conditions. Larvae were fed daily on larval food (a mixture of brewer’s yeast (Vital Health Foods, South Africa) and finely ground dog biscuits (West’s traditional crunching biscuits treats, Martin and Martin, South Africa) prepared at a ratio of 1:3). All adults were maintained on a 10 % sucrose solution soaked into cotton pads and were provided with a blood meal twice weekly.

### Irradiations

All irradiations were carried in a Gammacell 220 (MDS Nordion, Ottawa, Canada). To ensure that test insects received the desired dose during irradiation, a thorough dose mapping of the irradiation chamber was first carried out. This led to the development of a nylon phantom that allows dose build-up and backscatter and a water filled target volume that ensures a dose variation of less than 6 %. In all cases, pupae were irradiated in batches of 250-500 suspended in 150 ml distilled water (dH_2_O).

### Experimental Procedures

**Fig. 1:**
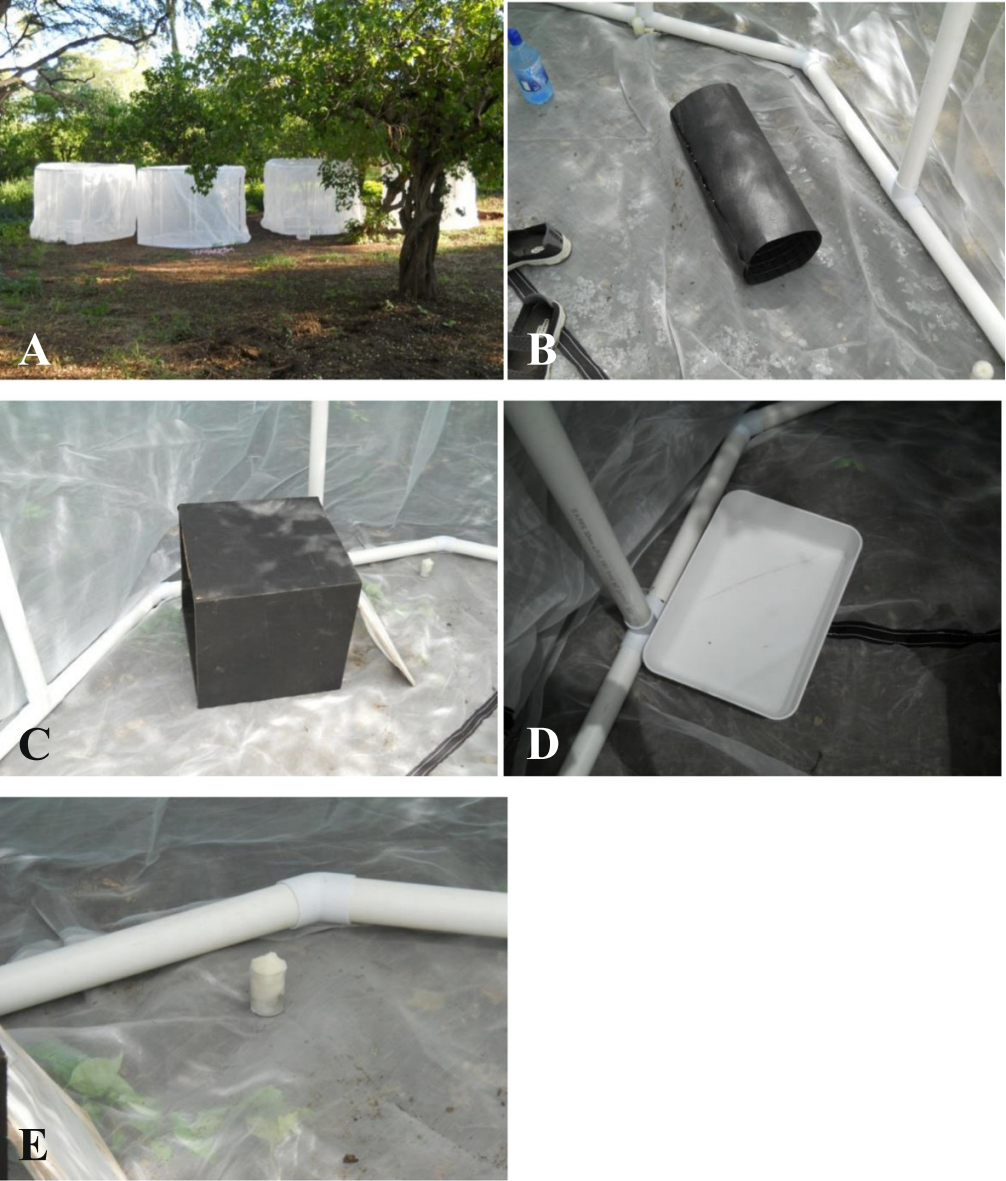
Experimental set-up of field cages during field competitiveness assays: **a** field cages placed under tree canopy cover which provided shading; (**b**) and (**c**) mosquito resting containers; **d** trays filled with water for additional humidity; **e** plastic jar with sucrose solution soaked cotton wool provided as an energy source for mosquitoes during assays
    The mosquitoes were released into semi-field cages at the following ratios: a) Control fertile: 200 fertile AMAL males + 200 fertile AMAL females; b) Sterile control: 200 irradiated GAMA males + 200 fertile AMAL females; c) Treatment 1:1:1: 200 irradiated GAMA males + 200 fertile AMAL males + 200 fertile AMAL females; d) Treatment 5:1:1: 1000 irradiated GAMA males + 200 fertile AMAL males + 200 fertile AMAL females; e) Treatment 10:1:1 2000 irradiated GAMA males + 200 fertile AMAL males + 200 fertile AMAL females. After release into cages mosquitoes were given four days of mating and all live females were recovered after the mating period. Procedures to determine insemination rates, fertility and fecundity were as described for laboratory competitiveness assays above. These experiments were repeated three times. In each replicate the position of each treatment/control was randomly selected.

(i) Dose optimisation
  To determine the optimal dose which induces sterility without compromising relative mating vigour, the effect of gamma irradiation on male pupae was tested at four different doses based on extrapolated data from dose-response curves for *An. arabiensis* [31].
  Male pupae were separated from female pupae manually based on pupal terminaliae morphology. After sex separation, male pupae aged 24-30 hrs were transferred in batches of 250-500 pupae suspended in 150 ml dH_2_O to an irradiation facility located approximately 800 m from the insectary. For each biological repeat, irradiation was carried out at the following range of irradiation doses [0 (controls), 70, 75, 80 and 100 Gy] using material from the same cohort. After irradiation, measures of adult emergence, longevity, fecundity and fertility were used to select the optimal dose.
  Emergence
    After irradiation, pupae were allowed to emerge into adults under standard insectary conditions in 30 × 30 × 30 cm BugDorm® insect cages (Megaview Science Education Services Co Ltd, Taiwan). The numbers of adults successfully emerging for each irradiation dose were recorded, as were those in the un-irradiated controls.
    After emergence, adults that accrued from each irradiation dose were pooled to get homogeneous samples before being separated into two groups. The first group was used for determining adult survivorship rates and the second group was used for fecundity and fertility studies.
  Adult longevity
    After irradiation, 50 randomly selected newly emerged males were placed in BugDorm® cages to assess longevity after exposure to the different doses. For each of the three replicates, two controls were set up. The first consisted of those adults from GAMA pupae which were handled as irradiated pupae, except that they remained un-irradiated (separation control). The second control consisted of newly emerged adults from the GAMA colony (non-separation control). The latter control was included to estimate the effect of physical stress on pupae due to physical handling during sex separation. Measurement of longevity of males from the baseline strain (AMAL), which was used to create GAMA, was not undertaken as this has previously been described [9]. All adults were maintained on 10 % sugar solution soaked into cotton wool under standard insectary conditions for the duration of the experiments. Survival was assessed daily until 100 % mortality was reached in all cages.
  Fecundity
    A total of 50 randomly selected, newly emerged irradiated GAMA males from each dose were allowed to mate with virgin females from the AMAL colony for four nights in BugDorm® cages. After four nights, all males were removed from cages and two blood meals were subsequently provided to the mated females over a five-day period. Two days after the second blood meal, each female was individually transferred to an oviposition glass vial to induce oviposition [32]. Eggs from each female were counted using a hand-held magnifying lens. For each biological repeat, three controls were set up consisting of (i) 50 virgin AMAL males mated with 50 virgin AMAL females (baseline control); (ii) 50 virgin GAMA males obtained from pupae that were un-irradiated (but otherwise handled as irradiated pupae) mated with 50 virgin AMAL females (separation control); and (iii) 50 newly emerged virgin un-irradiated GAMA males mated with 50 virgin AMAL females (non-separation control). The mean number of eggs/female laid was calculated. Female fecundity was compared between treatments and controls.
  Fertility
    For each treatment and the controls, eggs from individual females were transferred into plastic bowls (27 cm × 16 cm × 6.5 cm) containing 150 ml of distilled water and allowed to hatch. Hatch rates were then determined using procedures previously described by Munhenga et al. [9]. The mean number of days taken to hatch and proportion of hatched eggs was determined and compared between treatments and controls.
(ii) Mating competitiveness
  In order to assess the relative mating competitiveness of sterilized males and to measure the effect of increased release ratios on hatch rates, competitiveness assays were carried out under both laboratory and field conditions.
  Preparation of material for competitiveness assays
    Male (GAMA and AMAL) and female (AMAL) pupae were separated manually under a stereomicroscope. For field competitiveness assays, pupal separation was done daily for four consecutive days in order to accumulate large enough samples. Pupae were collected at specific times to control for age and were separated by gender. GAMA male pupae were irradiated when aged 24-30 h. Irradiation was achieved by exposing pupae to gamma rays generated by a cobalt-60 source using an extrapolated dose rate designed to give an irradiation dose of 75Gy. After manual separation, AMAL males and females were allowed to emerge normally without further treatment (fertile cohorts).
    All treatments (fertile males, sterile males and virgin females) were maintained in separate cages with sugar water provided *ad libitum* for 2 days (laboratory assays) and between 2-4 days following emergence in field assays to allow for sexual maturation.
  Competitiveness of irradiated GAMA males under laboratory conditions
    All laboratory competitiveness assays were carried out under standard insectary conditions of 25 °C and 85 % relative humidity in modified standard 30 cm × 30 cm × 30 cm BugDorm® insect cages. The cages were modified by scraping / sandpapering the inside of the cages to create a rough surface that facilitates improved tarsal cohesion during mosquito resting.
    To determine competitiveness of irradiated GAMA males under laboratory conditions, mosquitoes were released into BugDorm® cages in the following ratios. a) Control fertile: 50 fertile AMAL males + 50 fertile AMAL females; b) Sterile control: 50 irradiated GAMA males + 50 fertile AMAL females; c) Treatment 1:1:1: 50 irradiated GAMA males + 50 fertile AMAL males + 50 fertile AMAL females; d) Treatment 5:1:1: 250 irradiated GAMA males + 50 fertile AMAL males + 50 fertile AMAL females; and e) Treatment 10:1:1: 500 irradiated GAMA males + 50 fertile AMAL males + 50 fertile AMAL females. Mating was allowed for four nights to maximise the chances of successful mating. After the fourth night, females were re-collected using hand-held mechanical aspirators, and transferred to separate BugDorm® holding cages by treatment before being given two blood meals over a five day period. After the second blood feeding (two days after the first), 50 randomly selected gravid females were isolated from each respective cage and induced to oviposit using darkened 250 ml oviposition cups filled with approximately 150 ml of distilled water. Oviposited eggs from each cage were transferred onto a thin strip of 240 mm filter paper (Munktell Cat No. FLAS3206240) and counted under a hand-held magnifying lens. Mean numbers of eggs laid were calculated and compared between cages. Owing to variation in the number of egg batches produced by each female, fecundity was scored as the number of eggs laid by females per single gonotrophic cycle. After fecundity was determined, eggs from each treatment and control group were thoroughly mixed and a sub-sample of 100 eggs transferred into plastic bowls containing distilled water to allow hatching. Egg hatch rates were monitored as described in [9]. To determine insemination rates a sub-sample of 10 females was randomly selected and removed in order to determine the proportion/rate of insemination. Each female’s spermatheca was dissected and the presence of spermatozoa was assessed under a dissecting microscope (Wild, Heerbrugg M5-71661, Switzerland) at 200 X magnification. The proportion of inseminated females was calculated for each treatment and corresponding controls. All experiments were replicated three times.
  Competitiveness of irradiated GAMA males under semi-field conditions
    Field competitiveness assays were carried out during mid-summer (February) to late autumn (May), a period which normally coincides with the end of the rainy season. Temperature and humidity were monitored continuously using HOBO data loggers (Onset, Pocasset, MA). All evaluations were carried out in semi-field cages made from Anti-Thrip Netting (2.9 m diameter × 2.0 m high with floor) which allowed simulation of prevailing ambient weather conditions (Fig. 1A). Two types of mosquito resting surfaces/containers were placed in each cage. The first type was a 30 cm × 30 cm × 30 cm wooden resting box lined with black felt with one side having a hinged cover to allow mosquito access and covered with damp blankets to maintain a reasonable humidity, and the second was a cylindrical tube, 45 cm long by 15 cm diameter, made of black felt rolled around a black wire mesh (Fig. 1B and C). Four plastic jars with cotton pads soaked in 10 % sucrose solution were placed in each cage to provide mosquitoes with an energy source (Fig. 1E). For additional humidity, two trays filled with 2.5 L water were included (Fig. 1D).

### Parameters measured and data analysis

Data on numbers of females recovered after mating, fecundity, hatch rates, adult longevity and adult emergence were summarised. Data were first analysed for any statistical differences between replicates for each treatment. Unless otherwise stated, data from the same treatments between replicates were then pooled. After pooling data one-way ANOVA was used to analyze differences in means of each variable between treatments and controls. Percentage values for adult survivorship as well as adult emergence and insemination rates of females were checked for normality and transformed where applicable to achieve normal distribution. These were then compared for differences between treatment and control using ANOVA. Survival curves were analysed using Kaplan Meier survival analysis and Cox’s F test was used to compare mean difference in survivorship between treatments. Fertility (egg hatch rates) was calculated for each female population by dividing the number of first instar larvae by 100 randomly selected eggs for each treatment and controls. An average was calculated for each treatment and control and was used to test for statistical significance using one-way ANOVA. In addition, correlation analyses between dose and fecundity and fertility were performed. In all cases data were analysed in SPSS version 22 and a *P*-value of less than 0.05 was considered to indicate statistical significance. The competitive value and expected egg hatch rates were computed using methods described by Fried [33]. Induced sterility was calculated using the method described by Yamada [27].

## Results

### Dose optimisation

#### Emergence

Mean emergence rate significantly differed between treatments (One way ANOVA; F = 7.22, *p* < 0.05). Pair-wise comparisons showed that the adult emergence rate from pupae without extensive handling (non-separation control) was significantly higher compared to the other groups (Table 1).

**Table 1:** Mean percentage male adult emergence for *An. arabiensis* pupae irradiated at different doses and mean survival time of resultant adults reared under standard insectary conditions. Separation refers to pupal separation by gender

Treatment	Emergence		Adult survivorship	
	Total no. pupae induced to emerge	Mean % emergence ± SD (95 % CI)	Total no. adult males monitored	Mean survival time ± SE (95 % CI)
Non-separation control	447	96.8 ± 2.6 (94.1 - 99.6)^a^	132	13.5 ± 0.79 (11.9 - 15.0)^a^
Separation control	448	78.7 ± 6.9 (71.6 - 86.2)^b^	117	12.7 ± 0.76 (11.6 - 13.3)^a^
70Gy	447	80.7 ± 7.1 (73.2 - 88.1)^b^	142	12.6 ± 0.71 (11.2 - 14.9)^a^
75Gy	452	82.6 ± 4.7 (77.6 - 87.6)^b^	138	13.4 ± 0.63 (13.1 - 14.7)^a^
80Gy	449	83.4 ± 5.4 (77.7 - 89.1)^b^	136	10.2 ± 0.67 (9.0 - 11.5)^b^
100Gy	453	83.4 ± 5.4 (77.8 - 89.0)^b^	136	10.3 ± 0.29 (9.0 - 11.6)^b^

Within columns, values followed by different lower case letters are statistically different (*P* < 0.05; one way ANOVA)

#### *Longevit***y**

Data on mean survival time of adult males emerging from irradiated pupae and corresponding controls are summarised in Table 1. Males drawn directly from the GAMA colony that were not extensively handled (non-separation control) showed the highest survival rate. Log Rank (Mantel-Cox) comparison of survival rates showed that there is a significant difference in survival between the samples (Chi-square = 35.07, DF = 5, *P* < 0.05). Following a pair wise comparison, results showed that adults irradiated at the higher irradiation doses (80 or 100 Gy) showed significantly reduced longevities compared to the other groups.

#### Fecundity

Fecundity data of AMAL females mated with irradiated males and controls are summarised in Table 2. The mean percentage of females successfully ovipositing eggs ranged from 37 - 58 % in the controls and 23 – 33 % in the treated cohorts. However, there was not statistically significant difference in the number of females laying eggs between those females mated with unirradiated males (controls) and those mated with irradiated males (treatments); (one way ANOVA, F = 1.14; *P* = 0.38). Despite irradiation treatments, no significant differences were detected in egg production between treatments and control groups (one way ANOVA, F = 1.71; *P* = 0.12).

**Table 2:** Fecundity and fertility of *An. arabiensis* females mated with males irradiated at different doses and their corresponding controls

Treatment	Mean % of females induced to lay eggs	Mean % of females laying eggs ± SD	Mean no. of eggs laid per female ± SD (95 % CI)	Mean % egg hatch rates ± SD (95 % CI)
Baseline Control	21.7	57.7 ± 6.4^a^	66.3 ± 48.6 (49.9 - 82.7)^a^	64.9 ± 34.0 (51.2 - 78.6)^a^
Non-separation Control	22.7	40.4 ± 24.8^a^	48.5 ± 31.1 (35.6 - 61.3)^a^	28.8 ± 13.9 (18.1 - 39.5)^b^
Separation Control	21.7	37.4 ± 28.8^a^	57.2 ± 41.2 (38.9 - 75.5)^a^	26.7 ± 13.9 (20.0 - 33.4)^b^
70 Gy	20.3	32.8 ± 23.6^a^	76.7 ± 67.2 (46.1 - 107.3)^a^	1.5 ± 1.9 (0.3 - 2.7)^c^
75Gy	21.3	23.6 ± 5.6^a^	49.4 ± 39.5 (27.5 - 71.3)^a^	0.6 ± 1.1 (0.3 - 1.6)^c^
80 Gy	22.3	23 ± 15.7^a^	50.1 ± 39.6 (28.1 - 72.0)^a^	0.9 ± 1.9 (0.5 - 2.4)^c^
100 Gy	20.7	28.6 ± 18.8^a^	36.7 ± 38.3 (16.3 - 57.1)^a^	0.5 ± 1.1 (1.1 - 2.2)^d^

Within columns, values followed by different lower case letters are statistically different (*P* < 0.05; one way ANOVA)

NB: Baseline control refers to newly emerged AMAL females mated with GAMA males without any handling at the pupal stage. Non-separation control refers to newly emerged unirradiated GAMA males mated with fertile AMAL females. Separation control refers to unirradiated GAMA males mated with fertile AMAL females after they were separated manually at the pupal stage and transported to the irradiation facility and back to the insectary

#### Fertility

Egg hatching rates from females mated with irradiated males and corresponding controls are summarised in Table 2. AMAL females mated with fertile wild-type AMAL males showed the highest mean successful hatch rate at 47.11 % (95 % CI: 33 % - 61.3 %). The lowest hatch rate of 0.12 % (CI; 0.14 % - 0.4 %) was recorded in eggs from females mated with GAMA males irradiated at 100Gy. There was a statistically significant difference in hatch rates between treatments and controls (one way ANOVA; F = 16.9; *P* < 0.05). Correlation analysis showed that there was a significantly negative relationship between the dose received and subsequent mean percentage egg hatch rates (R^2^ = 0.6; *P* = 0.04).

### Mating Competitiveness

#### Recovery of females

Some test mosquitoes died (natural rate of mortality) during the four days mating period, precluding the recovery of all test females. The average percentage of females recaptured from semi-field cages was 59.9 % with a minimum and maximum recovery rate of 24 % and 96.6 % respectively (Table 3). Overall, there was no significant difference in the percentages of females recaptured from the treatment and control cages (One-way ANOVA, F = 2.87; *P* = 0.07). However, during the first replicate there was a very low female recovery (24 %) in the 10:1:1 treatment cage and this was attributed to an ant invasion. For subsequent trials the cages were placed on top of large plastic sheets which prevented ants moving into the cages.

**Table 3:** Mean percentage insemination rates and fecundity of *An. arabiensis* AMAL females following mating competitiveness experiments under semi-field and laboratory conditions

Treatment (sterile males: fertile males: fertile females)	Field assays			Laboratory assays	
	Mean % of females recovered ± SD (min - max)	% Insemination rate ± SD	Fecundity^#^ (Mean no. eggs produced/female ± SD)	% Insemination rate ± SD	Fecundity (Mean no. eggs produced/female ± SD)
0:1:1	56.1 ± 20.6 (43 - 79.9)^a^	88.3 ± 10.4^a^	32.5 ± 21.3^a^	95 ± 0.0^a^	7.5 ± 1.3^a^
1:0:1	63 ± 11.8 (55 - 76.5)^a^	89.7 ± 10.0^a^	9.2 ± 4.4^a^	98.3 ± 2.9^a^	5.7 ± 2.9^a^
1:1:1	59.8 ± 16.9 (49.5 - 79.3)^a^	93.3 ± 7.6^a^	15.8 ± 8.9^a^	100^a^	11.8 ± 10.3^a^
5:1:1	63.5 ± 20.0 (51.5 - 86.6)^a^	90.0 ± 13.2^a^	25.4 ± 15.3^a^	98.3 ± 2.9^a^	8.1 ± 7.0^a^
10:1:1	57.2 ± 36.7 (24 - 96.6)^a^	93.3 ± 5.8^a^	26.6 ± 13.3^a^	98.3 ± 2.9^a^	15.5 ± 12.2^a^

^#^determined by mass egg plating. Irradiation of males was carried out at 75 Gy during field assays and 70 Gy during laboratory assays. Within columns, values followed by different lowercase letters are statistically different (*P* < 0.05; one-way ANOVA)

For the laboratory competitiveness assays, the average percentage of females recovered after four days of mating ranged from 89 - 100 %. There was no statistically significant difference in female recovery between treatment and control cages (One way ANOVA, F = 1.83; *P* = 0.2).

#### Insemination and fecundity

Data for insemination rates and fecundity for females from control cages and treatment cages are summarised in Table 3. Results showed that there was no significant difference in insemination rates between control and treatment cages for both field and laboratory assays (One-way ANOVA: F = 0.16, *P* = 0.95 (field assays) and F = 2.0, *P* = 0.17 (laboratory assays)). Although mean numbers of eggs produced by females mated with irradiated males only (sterile control) during field competitiveness assays were lower than in other treatments, no statistically significant differences were detected due to high variability between replicates (one way ANOVA, F = 1.34, *P* = 0.32). However, there was a positive correlation between the number of eggs produced per female and the number of available males per cage (R^2^ = 0.7; *P* = 0.02). There was no statistically significant difference in the number of eggs produced per female between controls and treatments (One way ANOVA; F = 0.96; *P* = 0.456).

#### Competitiveness index and induced sterility

Results of mating competitiveness experiments conducted under both laboratory and natural conditions in semi-field cages are presented in Table 4 and Table 5. The competitiveness values and expected egg hatch rates were computed using Fried [33] equations. Induced sterility in each cage was calculated using the method described by Hanano et al. [27]., Results showed that for mating ratios of irradiated male: normal male: normal female of 0:1:1, 1:0:1, 1:1:1, 5:1:1 and 10:1:1, the average egg hatch rates were 89, 3, 77, 63 and 44 % respectively under laboratory conditions. Similarly, the mean egg hatch rates during field competitiveness assays reduced with increasing proportions of irradiated males. There was a marked statistical difference in egg hatch rates between controls and treatments for both field and laboratory competitiveness assays (One-way ANOVA: F = 12.2, *P* < 0.05 (field assays) and F = 13.1, *P* < 0.05 (laboratory assays)). The competitiveness values recorded from the treatment cages at ratios of 1:1:1, 5:1:1 and 10:1:1 (irradiated male: normal male: normal females) were higher in the field tests compared to the laboratory tests, ranging from 0.29 to 0.36 in field tests and 0.08 to 0.16 in laboratory tests. The induced sterility in the 1:1:1; 5:1:1 and 10:1:1 cage was 13.5, 29.2 and 50.6 respectively in the laboratory tests. The induced sterility was comparatively higher at 25.8, 58.4 and 73 % for the 1:1:1; 5:1:1 and 10:1:1 cages respectively in the field tests.

**Table 4:** Mating competitiveness values for *An. arabiensis* GAMA males irradiated at 70 Gy competing with fertile *An. arabiensis* AMAL males for AMAL females under laboratory conditions in 30 cm × 30 cm × 30 cm bug-dorm cages

Treatment	S/N	Observed Hatch Rate ± SD (%)	Expected Hatch Rate (%)	Induced Sterility (%)	Competitiveness value
Fertile Control		89 ± 16.2			
Sterile Control		3 ± 2.5			
1:1:1	1	77 ± 17.9	46	13.5	0.16
5:1:1	5	63 ± 21.6	17	29.2	0.08
10:1:1	10	44 ± 24.1	11	50.6	0.11
Average CI = 0.12					

S/N refers to the ratio of sterile to fertile males in each treatment cage

**Table 5:** Mating competitiveness values for *An. arabiensis* GAMA males irradiated at 75Gy competing with fertile *An. arabiensis* AMAL males for AMAL females under natural conditions in semi-field cages

Treatment	S/N	(%) Observed Hatch Rate ± SD	(%) Expected Hatch Rate	(%) Induced Sterility	Competitiveness value
Fertile control		89 ± 7.9			
Sterile control		1.3 ± 0.6			
1:1:1	1	66 ± 17.3	47.3	25.8	0.36
5:1:1	5	36.7 ± 5	16.8	58.4	0.29
10:1:1	10	23.7 ± 14.6	9.8	73	0.29
Average CI = 0.31					

S/N refers to the ratio of sterile to fertile males in each treatment cage

## Discussion

### Dose optimisation

Exposure of 24-30 h old male pupae to gamma rays ranging from 70 -100 Gy did not affect adult emergence. However, adult emergence data recorded in this study is lower than that recorded in the literature. In a life table analysis conducted during colonisation of AMAL in 2010, high emergence rates ranging from 90-96 % were observed [9]. Similarly, in dose optimisation experiments of an *An. arabiensis* strain from Sudan conducted by Helinski et al [31], adult emergence rates averaging 96 % were recorded. The low emergence rate recorded here may be attributed to the physical stresses imposed on the pupae during manual separation. The fecundity of females mated with irradiated males was generally not affected during dose optimisation experiments. The mean number of females laying eggs differed between those mated with irradiated males compared to those mated with fertile males. However, due to high data variability, this difference was not statistically significant. Females mated with irradiated males also laid fewer eggs compared to those mated with unirradiated males. There was a significant correlation between irradiation dose and fertility in which the higher the irradiation dose the higher the level of induced sterility. There was also an irradiation effect on longevity of males which was more pronounced at higher irradiation doses. This was probably due to high somatic damage at higher irradiation doses. Based on these results, it was decided that an irradiation dose of between 70-75Gy was sufficient to induce sterility in subsequent mating competitiveness experiments without affecting mating vigour. Such doses are in agreement with those reported elsewhere [27; 31]. These results also provided further evidence that adult survival and mating performance are negatively affected by high irradiation doses. Mosquito pupal emergence, survivorship and a low mating performance have previously been shown to be affected by prolonged exposure to ionising irradiation [29, 31]. These effects may be due to somatic cell damage as ionising radiation is non-specific [34].

### Mating competitiveness

The main concern with semi-field competitiveness assays is whether laboratory reared mosquitoes will survive long enough to mate under natural environmental conditions and adapt to continuous fluctuation in weather conditions. Colonisation is postulated to select for abnormal traits and modify colonised insects to behave abnormally under field conditions [22, 35]. Contrary to this it was observed that males were able to form mating swarms, similar to a study carried out in Sudan which showed that irradiated males which had been extensively handled through marking for a mark-release capture study actively participated in swarms and at times even initiated swarming [36]. Furthermore, the female recovery data after four days of mating suggests that mosquito survival during the semi-field assays was comparable to survival recorded during the laboratory assays, except in one instance when one of the cages was attacked by ants. Overall, extensive handling, transportation and variable temperatures during semi-field assays did not significantly stress mosquitoes or affect their mating vigour, as also evidenced by the high insemination rates recorded in samples of the recovered females. Additionally, there was no difference in the insemination capability of males mating under either laboratory or semi-field conditions and the insemination rates recorded compare well and in even surpass those previously recorded [27, 37, 38]. Another interesting observation was that females mated under semi-field conditions produced more eggs per female than those mated under laboratory conditions. This may be because the strain used for these studies is being constantly reinvigorated by field collected material and therefore shows a better mating performance under natural conditions than laboratory conditions. Revitalisation of colonies by wild-collected material is used in most SIT programmes to improve the mating ability of colonised strains [28].

### Competitiveness index and induced sterility

The competitiveness index (CI) gives an estimate of the competitiveness of irradiated males against their untreated counterparts [33] and induced sterility is a measure of the amount of sterility induced by treated males [27]. When used in parallel, the two values can be used to estimate a required inundative ratio in a planned or operational SIT programme. The competitiveness calculations from the experiments described here show that the competitiveness of irradiated GAMA males differs under laboratory and semi-field conditions. Irradiated GAMA males were only a tenth as competitive as AMAL males (CI = 0.1) under laboratory conditions. In contrast, irradiated GAMA males were substantially more competitive under semi-field conditions showing a CI of 0.3 meaning that a SIT programme using these males will need to inundate the target population by a ratio of three irradiated males to one wild fertile male in order to effectively reduced overall female fertility. The highest induced sterility (73 %) was obtained in the 10:1:1 field cage population. These results were unexpected as most studies have shown that irradiated laboratory males are normally more competitive under laboratory conditions than semi-field conditions. During laboratory and semi-field tests carried out in both small and semi-field large cages using *An. arabiensis,* the competitive index for small cages under laboratory conditions was approximately three times higher than that obtained in large cages under semi-field conditions [39]. As alluded to earlier, a possible explanation for these results could be the age of the colonies used for this study. AMAL was colonised in 2010 and is constantly being supplemented by wild field-collected stock while GAMA was developed by mating AMAL females with GSS ANO IPCL1 males and therefore contains the genetic background of AMAL. It is therefore possible that these strains still possess mating traits better suited to natural conditions. Another possible explanation is the size and type of cages used for laboratory competitiveness studies. AMAL adults are generally cultured in cylindrical 25 L cages whereas the mating experiments were carried out in cubic 27 L cages. This change may have affected the mating behaviour of AMAL females. The size of cages used may have negatively impacted mating especially at higher ratios in which the cages became overcrowded. As previously highlighted by Yamada et al. [27], competitiveness experiments cannot be compared between strains due to differences in strain types and experimental set-ups. However, the results of these semi-field assays tally with those obtained by Helinski et al. [39] where a CI value of 0.34 was obtained using irradiated *An. arabiensis* under semi-field conditions. Recently, Maiga et al. [38] reported a mean competitiveness factor of 0.53 in an *An. coluzzii* strain which has been under colony for six years. The competitiveness of colonised mosquitoes tends to show high variation between and within strains because of differences in radio-sensitivity and other biological factors. Hassan et al. [37] showed high CIs in irradiated and colony *An. arabiensis* males of 0.71 and 0.81 respectively. In the 1970s male mating competitiveness of the *An. albimanus* MACHO strain with similar chromosomal translocations as GAMA had a very high CI of 0.785 [28]. Chambers et al. [40] recorded a near equal mating competitiveness between male *Aedes polynesiensis* infected with *Wolbachia* competing with uninfected F_1_ males for F_1_ females under semi-field conditions, clearly showing that results on mating competitiveness cannot be transferrable between strains and highlights the importance of carrying out competitive tests for individual strains.

## Conclusions

Irradiation of *An. arabiensis* male pupae at dosages between 70-75Gy does not affect adult emergence and longevity and does not preclude males from mating. However, development, culturing and production processes (including strain introgression, irradiation, handling and transportation of mosquitoes) contributed to the significantly reduced mating competitiveness of irradiated males. A competitive index value of 0.31 was obtained. This means a sterile to wild type ratio of 3 to 1 need to be released in order to effectively reduce target population fertility under field conditions. These results will be used to inform subsequent small-scale pilot releases at selected sites as part of the ongoing feasibility assessments of the SIT as a complementary malaria vector control tool in South Africa. However, further developmental procedures are needed to improve the competitiveness of sterile GAMA males when competing against wild males for wild females.
